# Media and scientific communication about the COVID-19 pandemic and the repercussions on the population's mental health: A protocol for a systematic review and meta-analysis

**DOI:** 10.1097/MD.0000000000023298

**Published:** 2020-12-11

**Authors:** Isac Davidson Santiago Fernandes Pimenta, Ádala Nayana de Sousa Mata, Liliane Pereira Braga, Gidyenne Christine Bandeira Silva de Medeiros, Kesley Pablo Morais de Azevedo, Isaac Newton Machado Bezerra, Victor Hugo de Oliveira Segundo, Ana Clara de França Nunes, Gilberto Martins Santos, Suely Grosseman, Ismael Martínez Nicolás, Grasiela Piuvezam

**Affiliations:** a Department of Odontology, Post-Graduation Program in Public Health, Federal University of Rio Grande do Norte, Natal/RN; b Multicampi School of Medical Sciences of Rio Grande do Norte, Federal University of Rio Grande do Norte, Caicó/RN; c Department of Nutrition, Federal University of Rio Grande do Norte, Natal/RN; d Vitoria Academic Center, Federal University of Pernambuco, Vitoria/PE; e Department of Administrative Sciences, Federal University of Santa Maria, Santa Maria/RS; f Department of Paediatrics, Federal University of Santa Catarina, Florianopolis/SC, Brazil; g Department of Health Sciences, Catholic University San Antonio de Murcia, Murcia, Spain; h Department of Public Health, Federal University of Rio Grande do Norte, Natal/RN, Brazil.

**Keywords:** coronavirus disease 2019, health communication, media communication, mental health, systematic review

## Abstract

**Background:**

Good communication strategies are essential in times of crisis, such as the coronavirus pandemic. The dissemination of inaccurate information and the need for social isolation to control coronavirus disease 2019 (COVID-19) have shown a negative impact on the population, causing damage to mental health, with the appearance or worsening of symptoms of stress, fear, anxiety, and depression. Thus, the systematic review study is intended to gather evidence on the impact of information about COVID-19 on the mental health of the population.

**Methods:**

This systematic review protocol is conducted using the guidelines of the preferred reporting items for systematic reviews and meta-analyses protocols and the Cochrane Handbook for Systematic Reviews of Interventions. The review aims to include published studies that address the exposure of the general population to information about COVID-19, through observational and experimental studies, which consider the following outcomes: fear, stress, anxiety, and depression. Thus, a comprehensive research strategy will be conducted in the following databases: PubMed / Medline, Scopus, Web of Science, EMBASE, Science Direct, CINAHL, PsycINFO and Cochrane Central Register of Controlled Trials (CENTRAL). Two independent reviewers will perform all procedures, such as study selection, data collection, and methodological evaluation. Disagreements will be forwarded to a third reviewer. RevMan 5.3 software will be used for data analysis.

**Results:**

This systematic review will provide evidence of the influence of access to and consumption of media and scientific information about COVID-19 on the mental health of the population. It will consider information about the characterization of the study and the population studied, clinical and epidemiological information on mental health, and data on access to and consumption of media and scientific information.

**Discussion:**

The results should inform about the consequences of communication about the new coronavirus on the emergence or worsening of psychological and psychiatric symptoms, allowing to develop strategies to achieve effective communication of information to promote the mental health of the population.

**Systematic review registration number:**

PROSPERO CRD42020182918

## 1 Introduction

The widespread access of the population to the internet has increased the use of social media for health issues, which can contribute to the empowerment of patients and provide a closer relationship with health professionals. However, the use of social media not only provides benefits, it can be a source of inaccurate information that is not based on the science.^[1]^

In situations such as the coronavirus disease 2019 (COVID-19) pandemic, social media can be useful in updating the relevant guidelines for promoting health and controlling the virus, while inaccurate information or covering distressing events have proved to be harmful to the population.^[2]^

In this respect, experts and governments in some countries have failed to communicate the risks of exposure to the new coronavirus, sometimes using explanations that are not comprehensible and have no scientific basis on aspects related to epidemiology, and prevention and cure measures. Communications of this nature have reliability as their basic premise, with the objective of developing effective strategies for managing the pandemic and protecting public health. Thus, in situations where the information conveyed is ambiguous or the communication behaviors are inadequate, people develop different perceptions of risk and knowledge, levels of tolerance, and inappropriate fears.^[3]^

Information on the COVID-19 pandemic, on the disease, the means of transmission, and possible treatments is generated daily due to the fact that this is a new strain of coronavirus, as well as frequent updates from health agencies on the numbers of cases and deaths worldwide. The novelty of this topic drives individuals and society to seek information and updates in order to protect and conserve lives, considering that until the present time (July 2020), there is no cure or vaccine for the disease. This relentless drive to search for information on the unknown can generate a number of concerns for everyone in the community.^[2,4]^

Internet searches for updates on COVID-19 have increased from 50% to 70% for all age groups. According to the World Health Organization, the new coronavirus has been accompanied by an infodemic, that is, an overabundance of information, in which misinformation, rumors, and the manipulation of information can circulate and be absorbed very quickly. With a view to responding immediately to information received, there is sometimes no time to analyze the evidence carefully, which can interfere with people's behavior, affecting decision-making processes, possibly leading to risky behavior. People may also feel overwhelmed, emotionally drained, and unable to meet important demands.^[5]^

The urgency to produce and disseminate updated information on COVID-19 has led to the publication of articles, often without adequate review, to meet the commitment to the rapid dissemination of new information, which in some cases has led to the publishing of data with low scientific reliability.^[6]^ Misinformation and false reports about the disease are widespread on social media, and have contributed to increasing the level of stress, feeding the fears of those who access this information, and causing mental health problems.^[7]^ In addition, the death and loss of family members, and the dissemination of repeated images of seriously ill people, the bodies of the deceased, and coffins increase the fear, and further contribute to the anguish of the population.^[8]^

In a survey conducted by the Kaiser Family Foundation,^[9]^ 45% of American adults reported that their mental health was negatively affected due to concerns and stress about the virus, increasing the burden on the population's mental health, while control measures have been recommended, such as closure of establishments and social distancing. In the first weeks of the COVID-19 outbreak, a study conducted in China indicated that the population was already experiencing psychological distress in the early stages of the epidemic.^[10]^ Mazza et al^[11]^ observed a higher percentage of people with high and very high levels of distress in Italy during the pandemic when compared to previous European epidemiological statistics.

In Spain, a investigation of psychological symptoms during lockdown showed that participants reported symptoms of depression (27.5%), anxiety (26.9%), and stress (26.5%).^[12]^ The authors expressed their concern about the indiscriminate circulation of alarming videos about COVID-19, which may contribute to the psychological vulnerability of individuals.^[12]^ The study by Gao et al^[7]^ indicates that exposure to social media is frequent in 82% of the studied population, and is associated with increased risk of depression, as well as having a high association with anxiety symptoms and the combination of anxiety and depression.

Given the above, it is relevant to carry out a synthesis of scientific evidence on the impact of the media and rapid communication during the COVID-19 pandemic on individual's mental health, revealing the psychological impact and the way such information is communicated. This synthesis may contribute to establishing guidelines for better care of the population's mental health and guidelines for good communication strategies.

## 2 Objective

Describe a protocol of a systematic review that aims to identify the impact of the media and scientific communication on COVID-19 on the mental health of the population, as well as to identify the media and scientific communication strategies that are effective in promoting the population's mental health.

## 3 Methods and analysis

### 3.1 Study registering and reporting

The systematic review study is registered with PROSPERO, the international prospective register of systematic reviews, under number CRD42020182918. This systematic review protocol is based on the preferred reporting items for systematic reviews and meta-analyses protocols guidelines.^[13]^ Possible changes to the protocol will be described in the publication of the final report, which should be developed in accordance with the preferred reporting items for systematic reviews and meta-analyses^[14]^ and the Cochrane Handbook for Systematic Reviews of Interventions.^[15]^

### 3.2 Study selection criteria

#### 3.2.1 Type of studies

Observational studies (cross-sectional, case-control, cohort) and experimental studies (community trials, randomized, and nonrandomized clinical trials) will be included.

#### 3.2.2 Type of participants

Participants will be taken from the general population, exposed to information on COVID-19, who have been subject or not to intervention programs.

#### 3.2.3 Type of interventions

For the systematic review, interventions should aim to expose the groups’ information about COVID-19. In this review, interventions designed to investigate communication and the effect on the population's mental health should consider the time of exposure, and the selection of sources of information associated with improving the population's mental health.

#### 3.2.4 Types of outcomes

The results can consider the following health outcomes: fear, stress, anxiety, and depression.

### 3.3 Search strategy

This review should summarize the evidence published in primary studies, by searching the following databases: PubMed / Medline, Scopus, Web of Science, Science Direct, CINAHL, PsycINFO and The Cochrane Central Register of Controlled Trials (CENTRAL), and EMBASE. The search strategy must result from the combination of terms from the Medical Subject Title (MeSH) and the Health Sciences Descriptors (DeCS), considering the following groups of words:

Group 1: communication; communications media; mass media; social media; information dissemination; information technology; health communication.

Group 2: COVID-19; coronavirus; 2019-nCoV; SARS-CoV-2.

Group 3: mental health; fear; stress; post-traumatic stress disorder; anxiety; anxiety disorders; depression; depressive disorder.

The search terms used for the formation of the search equations will be combined with specific filters from each database. There will be no time and language limitations on the surveys to be carried out.

### 3.4 Study selection

**Figure 1:**
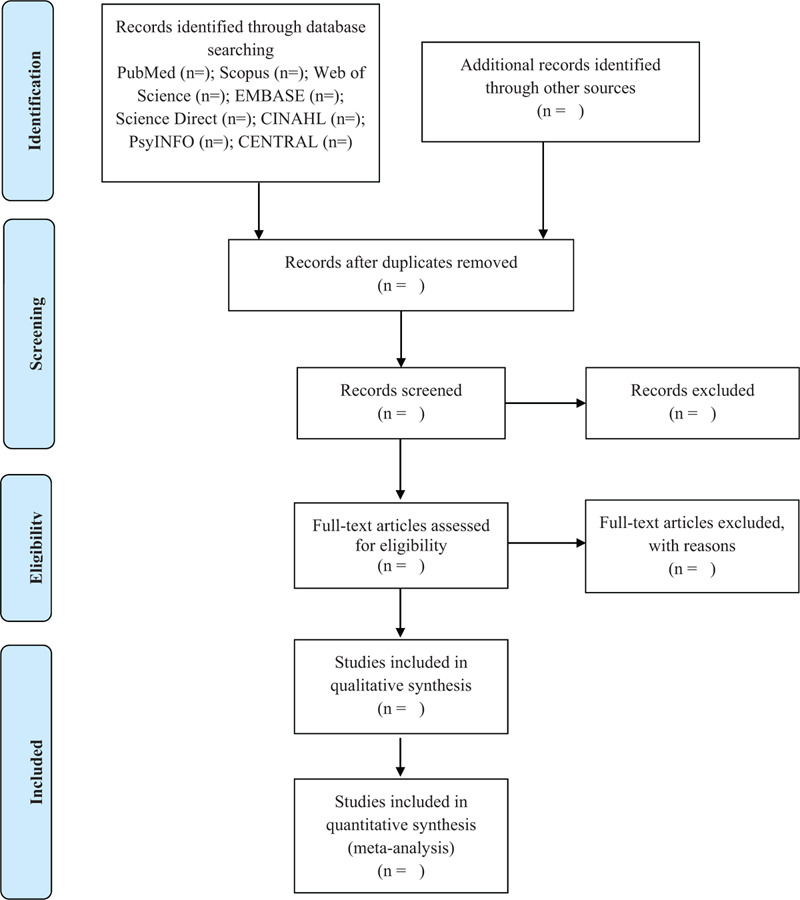
Flow diagram of study selection process.

Two reviewers will select the studies independently by reading the titles and abstracts, and then reading the full texts, according to the eligibility criteria, using the Rayyan systematic review application^[16]^ and reference management software. Any disagreements will be resolved by consulting a third reviewer. The references cited in the articles will be analyzed later to find other relevant articles not retrieved in the main search. Any disagreements regarding the selection of studies will also be resolved by consulting a third reviewer. The flowchart of the study selection process is shown in Figure 1.

The gray literature will be considered for the analysis of relevant information, such as the synthesis of protocols or critical analysis of the evidence, which may support the construction of guidance products about the communication of news and the repercussions for the mental health of the population.

#### 3.4.1 Inclusion criteria

Studies should consider, as inclusion criteria, publications that address the general population, regardless of characteristics such as sex, age, and exposure to COVID-19.

#### 3.4.2 Exclusion criteria

Animal studies will be excluded.

### 3.5 Data collection and management

The collection of all data will be done in a standardized manner by 2 authors independently, creating a database in a pre-designed spreadsheet and previously tested in the Excel program. The data collected will include relevant information on the identification of the studies (first author, year of publication, study period, sample size, study methods, geographic region); population characteristics (age, sex, period, and duration of recruitment); and clinical and epidemiological information (indexes of fear, anxiety, depression, and stress) and data on access and consumption of media and scientific information (means of communication, frequency of exposure, and evaluation strategies).

### 3.6 Dealing with missing data

If the data collected are not clear or incomplete, we will try to obtain the missing data by contacting the authors or co-authors of the article or the correspondents, by phone or e-mail. If we do not receive the necessary information, the data will be excluded from our analysis and will be covered in the Discussion section.

### 3.7 Risk of bias assessment

Two authors will independently assess the risk of bias for each included article, based on the Cochrane Handbook for Systematic Reviews of Interventions,^[15]^ to assess random sequence generation, allocation concealment, blinding, and evaluation of outcome data. In addition, incomplete results data, selective reporting, funding, and potential conflicts of interest associated with individual trials will also be considered. The risk of bias will be classified using predetermined criteria as follows: low, high, or unclear. The reviewers will be previously trained and their work calibrated to ensure uniformity in the evaluation of the criteria, and the Kappa index will be applied for agreement analysis.

### 3.8 Data synthesis

The data will be analyzed qualitatively, through a narrative synthesis. The data collected from the published studies must be standardized for comparability. A summary table will be produced, summarizing the data from the included studies. All data will be analyzed using Review Manager software (RevManV.5.3.3). For dichotomous results, we will derive the OR and 95% CI for each study. The heterogeneity between the results of the study will be assessed using a standard X^2^ test with a significance level of *P* < .05 and the I^2^ statistic, which is a quantitative measure of inconsistency between studies, with a value of 0% indicating no observed heterogeneity, until 50%, indicating moderate levels and, 75% or higher indicating substantial levels. If there is heterogeneity, a random-effects model will be used to combine the tests to calculate the relative risk (RR) and the 95% IC, using the DerSimonian-Laird algorithm in the Meta-analysis Package for R. Other characteristics and results of the study will be summarized narratively if a meta-analysis cannot be performed for all or some of the included studies. If possible, funnel plots will also be used to assess the presence of possible reporting biases and a linear regression approach will be used to assess the asymmetry of the funnel plot.

### 3.9 Dissemination and ethics

The results of this systematic review will be published in newspapers, conferences, or peer-reviewed journals. Ethics committee approval is not required, as this document does not involve individual patient data.

## 4 Discussion

World Health Organization has declared the outbreak of the new coronavirus to be a Public Health Emergency of International Concern – the organization's highest alert level, and seeks to unify efforts by all nations to halt its transmission. In addition to the development of research for the development of vaccines and treatment, it has been dedicated to monitoring and sharing expert information for the population and for collaboration in decision-making by governments and health institutions.^[17]^

In this same perspective, in the face of the widespread transmission of the virus, and its impact on the health of the population and on health systems, the scientific community responded quickly, promptly producing studies, as well as expanding the evaluation perspectives to understand more about the transmissibility, severity, consequences, and other resources associated with COVID-19.^[18]^ However, some studies have been contested, due to inadequate evaluations or conflicts of interest of researchers, which undermines effective and reliable scientific communication.^[6]^

The dissemination of inaccurate scientific information, coupled with misleading rumors and “conspiracy theories” that grew exponentially after the onset of the disease have become sources of fear, prejudice, and inappropriate behavior by the population, such as stockpiling groceries and personal protective equipment, like masks. Specifically in the context of the COVID-19 crisis, social media plays an important role in the dissemination of information. Erroneous information about the outbreak was also quickly disseminated, causing confusion, panic, and fear in the population, and making it difficult to build strategies and appropriate responses to cope with the pandemic.^[19]^

Xiang et al^[20]^ state that a clear communication process, with regular and accurate updates on the disease, should be provided to healthcare professionals and patients in order to minimize feelings of uncertainty and fear. The authors report that in previous SARS outbreaks, people reported various psychiatric morbidities, such as depression, anxiety, panic attacks, psychomotor agitation, and even suicide. In this sense, inadequate information and the social distancing strategy adopted for controlling COVID-19 can increase patients’ anxiety and guilt about the effects of infection, quarantine, and stigma on their families and friends, acting as barriers to appropriate medical and mental health interventions.^[20]^

In this context, it is necessary to identify the relationship between the consumption of information and the appearance and / or worsening of psychological symptoms, as well as to map strategies to achieve effective communication of information based on scientific evidence. Through the construction of a synthesis of scientific evidence, measures to help this problem can be formulated, targeting society in general, and specifically, the health organizations and professionals.

## Author contributions

**Conceptualization:** Isac Davidson Santiago Fernandes Pimenta, Ádala Nayana de Sousa Mata, Liliane Pereira Braga, Kesley Pablo Morais de Azevedo, Suely Grosseman, Ismael Martínez Nicolás, Grasiela Piuvezam.

**Formal analysis:** Isac Davidson Santiago Fernandes Pimenta, Ádala Nayana de Sousa Mata, Isaac Newton Machado Bezerra, Victor Hugo de Oliveira Segundo, Ana Clara de França Nunes, Gilberto Martins Santos, Suely Grosseman, Ismael Martínez Nicolás, Grasiela Piuvezam.

**Funding acquisition:** Grasiela Piuvezam.

**Methodology:** Isac Davidson Santiago Fernandes Pimenta, Ádala Nayana de Sousa Mata, Liliane Pereira Braga, Gidyenne Christine Bandeira Silva de Medeiros, Kesley Pablo Morais de Azevedo.

**Project administration:** Ádala Nayana de Sousa Mata, Grasiela Piuvezam.

**Writing – original draft:** Isac Davidson Santiago Fernandes Pimenta, Ádala Nayana de Sousa Mata, Liliane Pereira Braga, Gidyenne Christine Bandeira Silva de Medeiros, Kesley Pablo Morais de Azevedo, Isaac Newton Machado Bezerra, Victor Hugo de Oliveira Segundo, Ana Clara de França Nunes, Gilberto Martins Santos, Suely Grosseman, Ismael Martínez Nicolás, Grasiela Piuvezam.

**Writing – review & editing:** Isac Davidson Santiago Fernandes Pimenta, Ádala Nayana de Sousa Mata, Liliane Pereira Braga, Gidyenne Christine Bandeira Silva de Medeiros, Kesley Pablo Morais de Azevedo, Isaac Newton Machado Bezerra, Victor Hugo de Oliveira Segundo, Ana Clara de França Nunes, Gilberto Martins Santos, Suely Grosseman, Ismael Martínez Nicolás, Grasiela Piuvezam.
